# ATF3 Promotes Solasonine‐Triggered Ferroptosis in Glioma Cells by Suppressing Glycolysis

**DOI:** 10.1002/cns.71226

**Published:** 2026-10-24

**Authors:** Mingbo Jia, Hexin Xiao, Minghao Shi, Yao Li, Yang Yang, Jianing Zhao, Zhuoyue Shan, Pengfei Ge, Liyan Zhao

**Affiliations:** ^1^ Blood Transfusion Department The Second Hospital of Jilin University Changchun China; ^2^ Department of Laboratory The Second Hospital of Jilin University Changchun China; ^3^ Department of Neurosurgery The First Hospital of Jilin University Changchun China

**Keywords:** endoplasmic reticulum stress, ferritinophagy, ferroptosis, glioma, glycolysis

## Abstract

**Background:**

Glioma cells are characterized by rapid proliferation, a high degree of invasiveness, and significant heterogeneity. Despite the application of various treatment regimens in recent years, the prognosis for glioma remains poor. The induction of ferroptosis in tumor cells has emerged as a promising therapeutic approach. Solasonine (SS), a steroidal alkaloid derived from plants of the Solanaceae family, has multiple antitumor effects. However, whether it can inhibit glioma growth and its mechanism of action have not been studied.

**Methods:**

The effects of SS on ferroptosis in glioma cells were analyzed by detecting the levels of ferrous ions, reactive oxygen species, and malondialdehyde. The role of activating transcription factor 3 (ATF3) in SS‐induced ferroptosis in glioma cells was analyzed using western blotting, immunofluorescence, and confocal microscopy. An in vivo mouse model of glioma was constructed to further investigate the effects of SS.

**Results:**

We found that SS inhibited the proliferation of glioma cells and promoted their ferroptosis. SS treatment increased the intracellular ferrous ion, malondialdehyde, and reactive oxygen species levels. It increased the intracellular hydrogen peroxide level by regulating ATF3 to inhibit glycolysis and suppress the antioxidant system. Increased hydrogen peroxide levels activated nuclear receptor coactivator 4–dependent ferritinophagy and increased intracellular iron levels. Furthermore, SS activated the protein kinase RNA–like endoplasmic reticulum kinase/ATF4 endoplasmic reticulum stress pathway by inhibiting glycolysis and promoted ATF3 expression. This process formed a positive feedback loop. In a murine model, SS significantly inhibited glioma cell growth by inducing ferroptosis.

**Conclusion:**

These results indicate that SS promotes glioma cell ferroptosis in vitro and in vivo by inhibiting glycolysis through ATF3.

## Graphical abstract

Solasonine inhibits glycolysis by increasing ATF3 expression, thereby increasing hydrogen peroxide levels, promoting ferritinophagy and lipid peroxidation, leading to ferroptosis. Furthermore, hydrogen peroxide activates endoplasmic reticulum stress, promoting ATF3 expression, and the whole process forms a positive feedback loop.

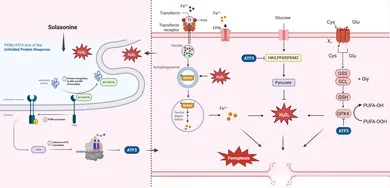

**Abbreviations**
3MA: 3‐methyladenine
4‐PBA: sodium phenylbutyrate
ATF3: activating transcription factor 3
ATF4: activating transcription factor 4
ATF6: activating transcription factor 6
ATG5: autophagy‐related protein 5
Baf‐A1: bafilomycin A1
DFO: deferoxamine
ER stress: endoplasmic reticulum stress
FAC: ferric ammonium citrate
FER‐1: ferrostatin‐1
FPN: ferroportin
FT: ferritin
FTH: ferritin heavy chain
GPX4: glutathione peroxidase 4
GRP78: glucose‐regulated protein 78
GSH: glutathione
H2A: histone H2A
IRE1α: inositol‐requiring enzyme 1α
LC3 I: microtubule‐associated protein 1 light chain 3‐I
LC3 II: microtubule‐associated protein 1 light chain 3‐II
MDA: malonaldehyde
NAC: N‐acetylcysteine
NCOA4: nuclear receptor coactivator 4
P62: sequestosome‐1
PERK: protein kinase R‐like endoplasmic reticulum kinase
PFKP: phosphofructokinase
PKM2: pyruvate kinase isozyme typeM2
ROS: reactive oxygen species
SS: solasonine
TF: transferrin
TFR: transferrin Receptor
XBP1: X‐box binding protein 1
xCT: solute carrier family 7 member 11

## 1 Introduction

Gliomas are the most common primary malignant tumors of the central nervous system, and their characteristics pose a challenge to treatment [1]. The proliferation and highly invasive growth of malignant glioma cells are the main reasons for the difficulty of complete glioma resection, high postoperative recurrence rates, and poor prognosis [2]. High‐grade glioma cells infiltrate surrounding normal brain tissue, resulting in indistinct boundaries that make precise intraoperative differentiation and resection difficult [3, 4]. Gliomas also exhibit cellular heterogeneity, with differences in radiation sensitivity; some radiation‐resistant tumor cells may survive and proliferate after radiotherapy, leading to tumor recurrence [5, 6, 7]. Due to the high chemotherapy resistance rate of malignant gliomas, the overall treatment effect is poor. The development of new and effective treatments for glioma is thus urgently needed [8, 9].

Solasonine (SS), a steroidal glycoalkaloid extracted from plants of the Solanaceae family, has a range of pharmacological effects, including antibacterial, anti‐inflammatory, and antitumor activities. It can inhibit tumor growth, invasion, and metastasis by interfering with the tumor cell cycle, inducing apoptosis and ferroptosis. It can also enhance the body's immune surveillance capabilities by regulating immune and inflammatory responses, promoting the recognition and elimination of tumor cells by immune cells [10, 11, 12, 13, 14].

The induction of ferroptosis, a form of cell death induced by elevated lipid peroxide levels that is dependent on iron overload, has been shown to effectively kill malignant tumor (lung cancer, liver cancer, and brain glioma) cells [15, 16, 17]. Two key factors in this induction are increases in the number of intracellular ferrous ions and the intensification of lipid peroxidation. Ferritinophagy involves the degradation of ferritin, the main intracellular iron storage protein, by enzymes in lysosomes, releasing stored iron and in turn affecting intracellular iron metabolism and oxidative stress levels [18, 19, 20]. In this process, nuclear receptor coactivator 4 (NCOA4) acts as a receptor, binding to ferritin and transporting it to autophagosomes, which subsequently fuse with lysosomes. Autophagy‐related protein 5 (ATG5) is a core protein in autophagy that is crucial for phagophore formation. During ferritinophagy, it participates in the extension and closure of the autophagosome membrane, helping to encapsulate ferritin and thereby promoting ferritin degradation and iron ion release [21]. Many studies have examined the roles of ferroptosis in the treatment of glioma; for example, ferroptosis has been shown to inhibit glioblastoma proliferation and the efflux of drugs from drug‐resistant cells, thereby reversing temozolomide resistance [22, 23, 24].

Activating transcription factor 3 (ATF3) has been shown to inhibit the expression of the key antioxidant proteins glutathione peroxidase 4 (GPX4) and xCT, and of catalase, which breaks down hydrogen peroxide into water and oxygen [25, 26]. It increases the intracellular hydrogen peroxide content by inhibiting glycolysis and the expression of antioxidant proteins. Hydrogen peroxide not only participates in ferroptosis by promoting an increase in ferrous ions [27], but also activates endoplasmic reticulum (ER) stress, of which ATF3 is a downstream target. This process leads to the elevation of intracellular reactive oxygen species (ROS) levels, exacerbating lipid peroxidation.

Even in the presence of ample oxygen, glioma cells tend to rely on glycolysis to provide energy for rapid tumor growth. The inhibition of glycolysis increases intracellular ROS levels [28], which can activate ferritinophagy [29]. Glycolysis affects ferroptosis in two main ways: by affecting the lactylation of ferroptosis‐related proteins through lactic acid and by altering the intracellular level of oxidative stress [30]. Thus, glycolytic pathway inhibition is considered to be a potential therapeutic strategy for glioma. The use of key glycolytic enzyme inhibition to inhibit glioma cell growth and proliferation has been explored [31]. The exploration of SS's mechanisms of action, and specifically how it induces ferroptosis in glioma cells, will aid the development of new targets for glioma treatment.

## 2 Materials and Methods

### 2.1 Cell Lines and Culture

U251MG (ECACC: 09063001) and T98G (ATCC: CRL‐1690) human glioma cells were cultured in Dulbecco's modified Eagle medium (Procell, China) containing fetal bovine serum (Procell) and penicillin/streptomycin (Beyotime, China). Cells in the logarithmic growth phase were used in the experiments.

### 2.2 Reagents and Antibodies

SS and cell counting kit‐8 (CCK‐8) kits were purchased from TargetMol (China). Ferrostatin‐1 (Fer‐1), deferoxamine (DFO), ferric ammonium citrate (FAC), liproxstatin‐1 (Lip‐1), adenosine triphosphate (ATP), bafilomycin A1 (Baf‐A1), 3‐methyladenine (3MA), N‐acetylcysteine (NAC), reduced L‐glutathione (GSH), sodium 4‐phenylbutyrate (4‐PBA), and sodium 2‐oxopropanoate were purchased from MedChemExpress (NJ, USA). Hydrogen peroxide was purchased from Millipore (MA, USA). Bicinchoninic acid (BCA) protein quantification kits were purchased from Thermo Fisher Scientific (Waltham, MA, USA). MitoSOX was purchased from Thermo Fisher Scientific (Waltham, MA, USA). Iron ion and pyruvate detection kits were purchased from Solarbio. ROS, malondialdehyde (MDA), ATP, GSH, and hydrogen peroxide detection kits were purchased from Beyotime. Transferrin receptor (TfR), transferrin, ferroportin (FPN), ferritin, GPX4, xCT, H2A, ß‐actin, ATG5, NCOA4, ferritin heavy chain (FTH), microtubule‐associated protein 1 light chain 3ß (LC3B), p62, pyruvate kinase M2 (PKM2), phosphofructokinase platelet type (PFKP), catalase, glucose‐regulated protein 78 (GRP78), protein kinase RNA–like endoplasmic reticulum kinase (PERK), ATF4, ATF3, and hexokinase 2 (HK2) antibodies were purchased from Abclonal (China).

### 2.3 Cell Treatments

U251MG and T98G cells were digested and seeded in a 96‐well plate (3000/well). At least three replicates each for the blank, control, and experimental groups were made. The cells were incubated in a culture incubator for 24 h to allow them to adhere. For cell viability assessment and half‐maximal inhibitory concentration (IC_50_) determination, SS (0, 5, 10, 15, 20, 25, 30, 35, 40, 45, and 50 μM) was added to the experimental replicates, and the samples were incubated for another 24 h.

To investigate whether SS induces ferroptosis in T98G and U251 cells, we pretreated the cells with the ferroptosis inhibitor DFO (500 μM) and the ferroptosis agonists FAC (500 μM), Fer‐1 (50 μM), and Lip‐1 (30 μM), respectively, for 1 h before SS treatment. In addition, we pretreated cells with GSH and its precursor NAC for 1 h. NAC increases cellular GSH levels, scavenges free radicals, and reduces oxidative stress damage. In a separate experiment, we administered the autophagy inhibitors 3MA (5 mM) and Baf‐A1 (1.5 μM) for 1 h before SS treatment. To explore the involvement of glycolysis, we pretreated cells with sodium pyruvate (SP, 10 mM) and ATP (2 mM) for 1 h. To explore whether hydrogen peroxide can regulate iron ions and ferritinophagy, we treated cells with 250 and 500 μM hydrogen peroxide for 8 h and with 500 μM hydrogen peroxide for 1, 2, 4, and 8 h. We also administered a combined NAC pretreatment for 1 h before SS treatment. To assess the involvement of ER stress, we pretreated glioma cells with 4‐PBA for 1 h.

### 2.4 Cell Viability Assays

For the cell viability assays, CCK‐8 solution (10 μL/well) was added to the cell samples, followed by gentle shaking to mix and incubation at 37°C for 1 h. To determine whether SS inhibits glioma cell proliferation, absorbance at 450 nm was then measured in a microplate reader (Thermo Fisher Scientific).

### 2.5 Cell Transfection

Lipo8000 transfection reagent (Beyotime), small interfering RNAs (siRNAs) targeting ATG5 (5′‐GACGUUGGUAACUGACAAATT‐3′) and ATF3 (5′‐GAUGAGAGAAACCUCUUUATT‐3′; GenePharma, China), and scrambled (negative control) siRNA (5′‐UUCUCCGAACGUGUCACGUTT‐3′) were prepared. The siRNAs (100 pmol) were added dropwise to the cell culture wells, followed by shaking to mix thoroughly. After transfection, the cells were incubated at 37°C and 5% CO_2_. After 4 h, the culture medium was replaced with complete serum to maintain normal cell growth.

### 2.6 Intracellular Marker Measurement

Because ferrous ion, MDA, and ROS levels are important markers of ferroptosis, we detected them in SS‐treated glioma cells with and without pretreatments or transfection at 6, 12, and 24 h. For ferrous ion detection, 200 μL lysis buffer was added to cell samples (2 × 106). An iron probe (10 μL) was then added, and the samples were mixed well. Absorbance at 593 nm was measured using a microplate reader. For MDA detection, the cells were lysed with radioimmunoprecipitation assay buffer, and 0.2 mL MDA detection working solution was mixed with 0.1 mL supernatant. The samples were centrifuged at 1000 × *g* and room temperature for 10 min, and 200 μL supernatant was then placed in a 96‐well plate. Absorbance at 532 nm was measured using an enzyme reader (Thermo Fisher Scientific).

Intracellular ROS expression was visualized using a fluorescence microplate reader (488 nm excitation, 525 nm emission; Thermo Fisher Scientific).

For pyruvate detection, lysis buffer was added to cell samples, the samples were centrifuged at 8000 × *g* for 10 min, the supernatants were collected, and 200 μL pyruvate detection reagent was added to the supernatant. Absorbance at 593 and 570 nm was measured using a microplate reader.

For the measurement of intracellular ATP, 100 μL lysis buffer was added to cell samples and the supernatants were collected. ATP detection reagent (200 μL) was added to the supernatants, followed by thorough mixing. ATP was detected using a chemiluminescence analyzer, and concentrations were calculated based on a standard curve. For the measurement of intracellular GSH/GSSG, the cells were washed with phosphate‐buffered saline (PBS) and centrifuged, 5,5′‐dithiobis‐(2‐nitrobenzoic acid) working solution and reaction buffer were added to the supernatant, and the samples were mixed well. The reaction system was incubated in a 37°C water bath or incubator for 30 min. Absorbance at 412 nm was detected using a microplate reader (Thermo Fisher Scientific). For hydrogen peroxide detection, 200 μL hydrogen peroxide detection reagent was added to supernatants, the samples were mixed well, and absorbance at 560 nm was measured using a microplate reader (Thermo Fisher Scientific).

### 2.7 Western Blotting

To comprehensively examine the mechanisms of action of SS on glioma cells, western blotting of proteins related to ferroptosis, ferritinophagy, glycolysis, and ER stress before and after treatment, pretreatment, and/or transfection was performed. Cell samples were lysed thoroughly with 200 μL lysis buffer, followed by centrifugation and supernatant collection. Protein concentrations were determined using a BCA kit. Protein samples were added to the gel wells, and electrophoresis separation was performed. Each gel and polyvinyl difluoride membrane was sandwiched in a transfer apparatus. The transfer buffer was used for the electrotransfer of the protein from the gel to the membrane. The samples were visualized using a chemiluminescence imaging system.

### 2.8 Confocal Microscopy

To explore the specific mechanism of SS‐induced ferroptosis, we detected the expression of ATF3 in the nucleus and cytoplasm by immunofluorescence staining and confocal microscopy (Zeiss, Baden‐Wurttemberg, Germany).

### 2.9 In Vivo Experiments

We established a murine model of glioma. The animal study was approved by the Ethics Committee of the First Hospital of Jilin University (Changchun, China, 20241230‐01). U251 cells (1 × 10^6^) in 100 μL PBS were injected subcutaneously into the right flanks of athymic BALB/c nude mice. There are 4 BALB/c nude mice in each group, totaling 12 BALB/c nude mice. Approximately 10 days later, the animals were divided into control (PBS), low‐dose (25 mg/kg) SS, and high‐dose (50 mg/kg) SS groups. The treatments were administered intraperitoneally once daily for 10 days. Tumor sizes were measured, and tumor volumes were calculated using the formula 0.5 × *a* × *B* ^2^, where *a* is the tumor length and *B* is the tumor width. On the second day after the final treatment, the animals were euthanized by cervical dislocation and the tumor tissue was excised. For the determination of GSH and GSSG, tissue samples are snap‐frozen in liquid nitrogen and subsequently ground into a powder. 100 μL of protein removal reagent is added to homogenize the sample; after centrifugation for 10 min, the supernatant is collected for the determination of total glutathione. Subsequently, GSH scavenging auxiliary solution and GSH scavenging working solution are added, and the mixture is incubated at 25°C for 60 min to detect GSSG; the GSH content is calculated using the formula: GSH = Total glutathione−(GSSG × 2). For the determination of ferrous ion, MDA, and hydrogen peroxide levels, lysis buffer is added to homogenize or lyse the tissue; following lysis, the sample is centrifuged, and the supernatant is collected for subsequent analysis. All of the aforementioned procedures must be performed at 4°C or on ice. The MDA, ferrous ion, hydrogen peroxide, and GSH/GSSG levels and protein expression in tumor tissue were analyzed as described above.

We established an orthotopic intracranial glioma model. The stereotaxic injection apparatus was pre‐warmed to match the body temperature of the NOD/SCID mice. The mice were anesthetized with isoflurane gas; following a local lidocaine injection into the scalp, a scalpel blade was used to expose the anterior and posterior fontanelles. After stereotaxic drilling of the skull, a flat‐tip microsyringe was used to inject a suspension containing 2 × 10^5^ U251 cells in 10 μL of PBS into the head of the caudate nucleus. The injection was performed at a rate of 1 μL per minute; upon completion of the injection, the needle was held in place for 5 min before being slowly withdrawn. The cranial burr hole was sealed with sterile bone wax, the skin incision was sutured, and the mice were placed in a temperature‐controlled incubator to recover. On the 7th day post‐surgery, the mice were further divided into three groups based on subsequent treatment regimens: a control group, a low‐dose treatment group, and a high‐dose treatment group. Intraperitoneal administration was employed, with doses of 25 mg/kg for the low‐dose group and 50 mg/kg for the high‐dose group; each group consisted of 4 NOD/SCID mice, totaling 12 mice. Each mouse received a single injection of 200 μL per day, undergoing a continuous treatment course with SS for 10 days. Upon completion of the drug administration period, the mice were euthanized via cervical dislocation, placed on ice, and the transplanted tumors were surgically dissected using sterile instruments. The specimen was then fixed with 4% paraformaldehyde, embedded in paraffin blocks, sectioned, and subjected to H&E staining and immunohistochemical staining.

### 2.10 Detection of Superoxide in Mitochondria

U251 and T98 cells were seeded into culture dishes and subjected to drug treatment and siATF3 transfection. A 5 mmol/L MitoSOX stock solution was prepared and subsequently diluted with serum‐free, high‐glucose DMEM medium to yield a 5 μmol/L MitoSOX working solution. Following the completion of the drug treatment, the cells were stained with the working solution under light‐shielded conditions. The 96‐well plates or culture dishes were then incubated in a cell culture incubator for 30 min, after which a confocal microscope was used to capture images of the cellular morphology, or a fluorescence microplate reader was employed to measure the fluorescence intensity of each group.

### 2.11 H&E Staining

Fresh tissue is fixed in formalin, embedded in paraffin blocks, and then sectioned into thin slices 3–5 μm in thickness. The sections are mounted onto glass slides and baked at 65°C for 2 h. The paraffin is first removed using xylene, followed by rehydration through a graded series of alcohols. Hematoxylin staining solution is applied for 5–10 min, after which—following a rinse in water—the cell nuclei appear bluish‐purple. The sections are briefly washed in acid‐alcohol for a few seconds to remove excess stain, then immersed in ammonia water or tap water to restore the blue coloration. Eosin staining solution is applied for 1–3 min to stain the cytoplasm red. The sections are then dehydrated through a graded series of alcohols and cleared in xylene until transparent. Finally, a drop of neutral mounting medium is applied, and a coverslip is carefully placed over the section, taking care to avoid air bubbles.

### 2.12 Immunohistochemical Staining

Paraffin sections need to be baked in a 60°C oven, then dewaxed with xylene, hydrated with a gradient of alcohols, and heated with sodium citrate retrieval solution. Endogenous enzymes are inactivated with hydrogen peroxide, and non‐specific sites are blocked with serum. Primary antibody is added first to bind the target protein, followed by secondary antibody to bind the primary antibody. A chromogenic agent such as DAB is added, and the enzyme catalyzes the production of a brown precipitate. Cell nuclei are counterstained with hematoxylin, dehydrated and cleared, and then mounted with neutral resin.

### 2.13 Statistical Analysis

All experiments in this study were performed in triplicate. The data were visualized and analyzed using GraphPad Prism 8.0 software, with the significance level set to *p* < 0.01. Results are expressed as means ± standard deviations. Statistical comparisons were made using one‐way analysis of variance.

## 3 Results

### 3.1 SS Inhibits Glioma Cell Proliferation and Promotes Ferroptosis

Relative to the control, SS treatment reduced glioma cell viability in a dose‐dependent manner, with a significant reduction observed at 35 μM (*p* < 0.01; Figure 1A). The IC_50_ values for SS for T98G and U251 cells were 36 and 35 μM, respectively. The concentration of 35 μM was used in subsequent experiments. DFO, Fer‐1, and Lip‐1 pretreatments inhibited SS‐induced cell death, whereas FAC pretreatment promoted it (all *p* < 0.01). In addition, SS treatment increased the intracellular ferrous ion, ROS, MDA, and GSH/GSSG levels, and the DFO, Fer‐1, and Lip‐1 pretreatments reduced them (all *p* < 0.01; Figure 1B–F). Pretreatment with GSH and NAC, respectively, also had protective effects in SS‐treated cells (Figure 1G). SS promoted the protein expression of transferrin and TfR, thereby increasing the intracellular ferrous ion and ferritin levels, and increased FPN protein expression in an attempt to accelerate iron ion efflux. At the same time, SS inhibited the expression of GPX4 and xCT, causing an increase in the intracellular ROS level (Figure 1H). These results demonstrate that SS induces ferroptosis in glioma cells by increasing the levels of ferrous ions, lipid peroxidation, and ROS.

**FIGURE 1:**
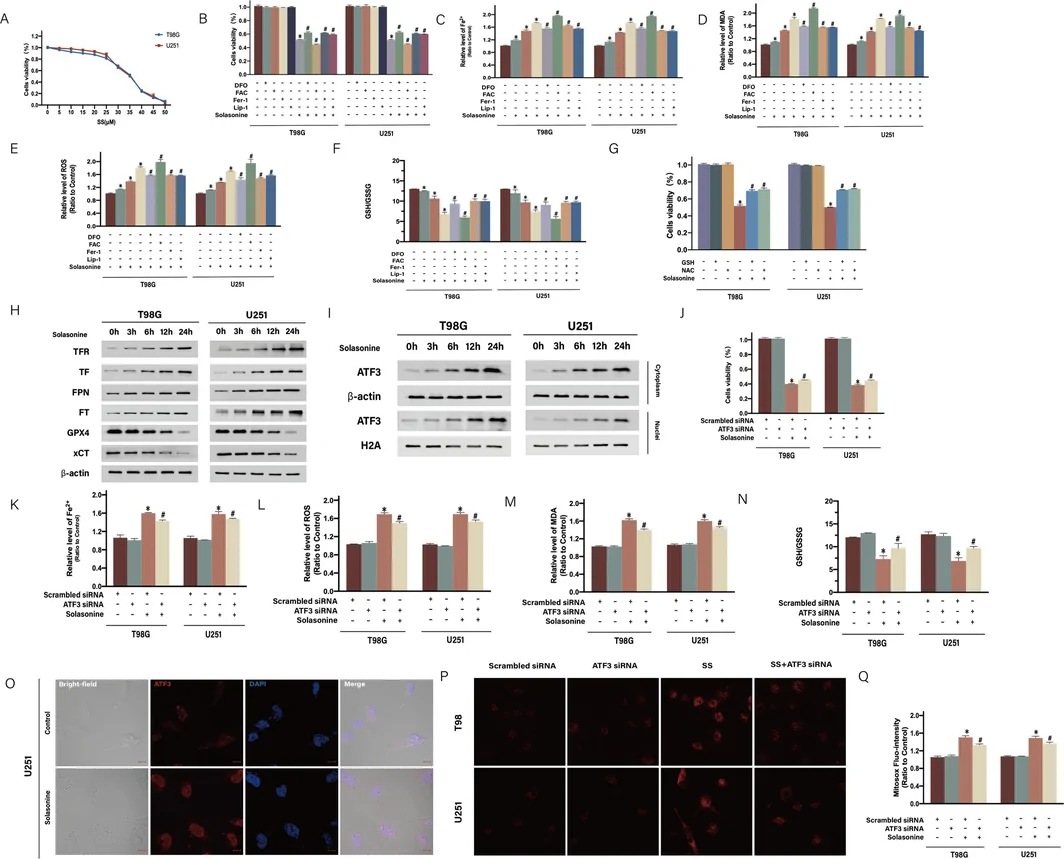
(A) Glioma cell survival at 24 h after SS treatment. (B) Glioma cell survival after pretreatment with DFO, FAC, Fer‐1, and Lip‐1, respectively, for 1 h before SS treatment. (C–F) Intracellular ROS, MDA, GSH/GSSG, and ferrous ion levels at 6, 12, and 24 h after SS treatment, with and without DFO, Fer‐1, Lip‐1, and FAC pretreatment for 1 h before SS treatment, respectively. (G) Glioma cell survival after SS treatment, with and without pretreatment with NAC and GSH for 1 h before SS treatment, respectively. (H) Western blots of ferroptosis‐related protein expression after SS treatment. Cell survival was assessed by the CCK‐8 assay. (I) Western blots of ATF3 expression in the cytoplasm and nuclei of SS‐treated cells. (J) Viability of cells treated with siATF3 followed by SS. (K–N) Ferrous ion, MDA, GSH/GSSG, and ROS levels in cells treated with siATF3 followed by SS. (O) Confocal microscopic images of ATF3 immunofluorescence in U251 cells treated with SS. (P, Q) Confocal microscopy images and fluorescence intensity measurements of mitochondrial superoxide in SS‐ and siATF3‐treated U251 and T98 cells. *p < 0.01 versus control, #*p* < 0.01 versus treatment group.

### 3.2 ATF3 Is Involved in SS‐Induced Ferroptosis in Glioma Cells

SS treatment increased ATF3 expression in the cytoplasm and nuclei of glioma cells (Figure 1I). Treatment with SS following siATF3 transfection resulted in increased cell viability, indicating that ATF3 promotes ferroptosis (Figure 1J). siATF3 transfection reduced the SS‐induced elevation of the ferrous ion, ROS, MDA, and GSH/GSSG levels (Figure 1K–N). ATF3 expression was increased, with translocation to the nucleus, in glioma cells treated with SS (Figure 1O). siATF3 transfection attenuated the ss‐induced elevation of mitochondrial superoxide levels (Figure 1P,Q). These results indicate that ATF3 is involved in the SS‐induced ferroptosis of glioma cells.

### 3.3 ATF3 Participates in SS‐Induced Glioma Cell Ferroptosis by Regulating Ferritinophagy

SS treatment increased the ATG5, FTH, and LC3‐II levels and decreased the NCOA4 and p62 levels, indicating that it promoted ferritinophagy (Figure 2A). 3MA and Baf‐A1 increased the survival rate of glioma cells and reduced the levels of ferrous ion, MDA, and ROS (Figure 2B–E). These autophagy inhibitors also suppressed the SS‐induced increase in ATG5 and ATF3 expression and decrease in FTH, p62, GPX4, and xCT expression (Figure 2F). These results suggest that ferritinophagy is involved in SS‐induced ferroptosis in glioma cells.

**FIGURE 2:**
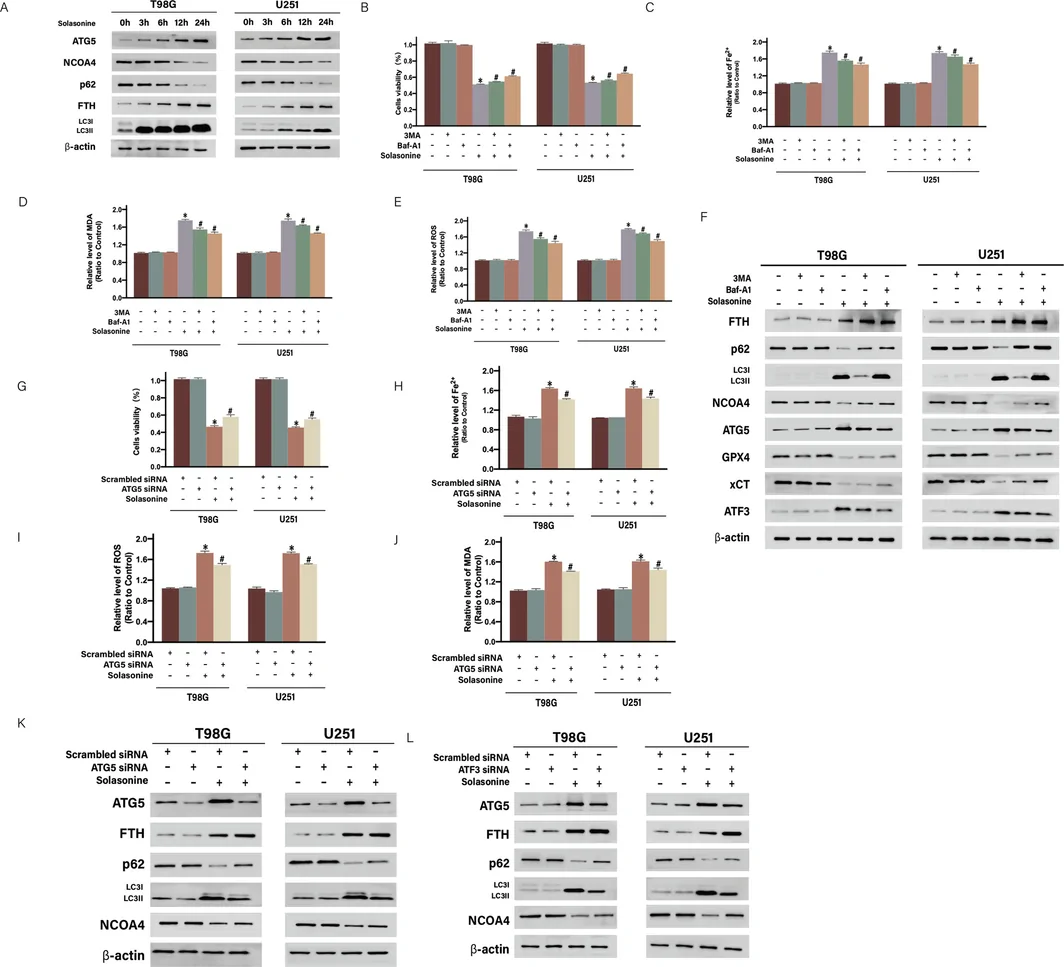
(A) Western blots of ferritinophagy‐related protein expression in glioma cells after SS treatment. (B–E) Cell viability and ROS, MDA, and ferrous ion levels after pretreatment with 3MA and Baf‐A1 followed by SS treatment. (F) Western blots of ferroptosis‐ and ferritinophagy‐related protein expression in glioma cells after 3MA and Baf‐A1 pretreatment and SS treatment. (G) Viability of glioma cells treated with siATG5 followed by SS. (H–J) ROS, MDA, and ferrous ion levels in glioma cells treated with siATG5 followed by SS. (K, L) Western blots of ferritinophagy‐ and ferroptosis‐related protein expression in glioma cells treated with siATG5 and siATF3, respectively, followed by SS. *p < 0.01 versus control, #*p* < 0.01 versus treatment group.

siATG5 treatment alleviated SS‐induced ferroptosis and decreased the ROS, MDA, and ferrous ion levels in glioma cells (Figure 2G–J). ATG5 and ATF3 knockdown each suppressed the increase in LC3‐II expression, promoted the increase in FTH expression, and suppressed the decrease in p62 and NCOA4 expression in glioma cells treated with SS alone, indicating that it inhibited ferritinophagy (Figure 2K). siATF3 treatment also reduced ATG5 expression (Figure 2L). Taken together, these results indicate that ATF3 promotes ferritinophagy by regulating ATG5, thereby increasing the ferrous ion level in glioma cells treated with SS.

### 3.4 ATF3 Promotes Ferritinophagy by Inhibiting Glycolysis and Increasing the Hydrogen Peroxide Level

The PKM2, PFKP, and HK2 levels decreased with increasing SS treatment time, indicating that SS inhibits glycolysis in glioma cells (Figure 3A). The SP and ATP pretreatments both increased the cell survival rate and decreased the ferrous ion, MDA, and ROS levels (Figure 3B–E), indicating that glycolysis is involved in SS‐induced glioma cell ferroptosis. These pretreatments also inhibited the SS‐induced increase in ATG5 and LC3‐II expression and decrease in FTH, p62, and NCOA4 expression, indicating that the regulation of ferritinophagy is the mechanism of this involvement (Figure 3F).

**FIGURE 3:**
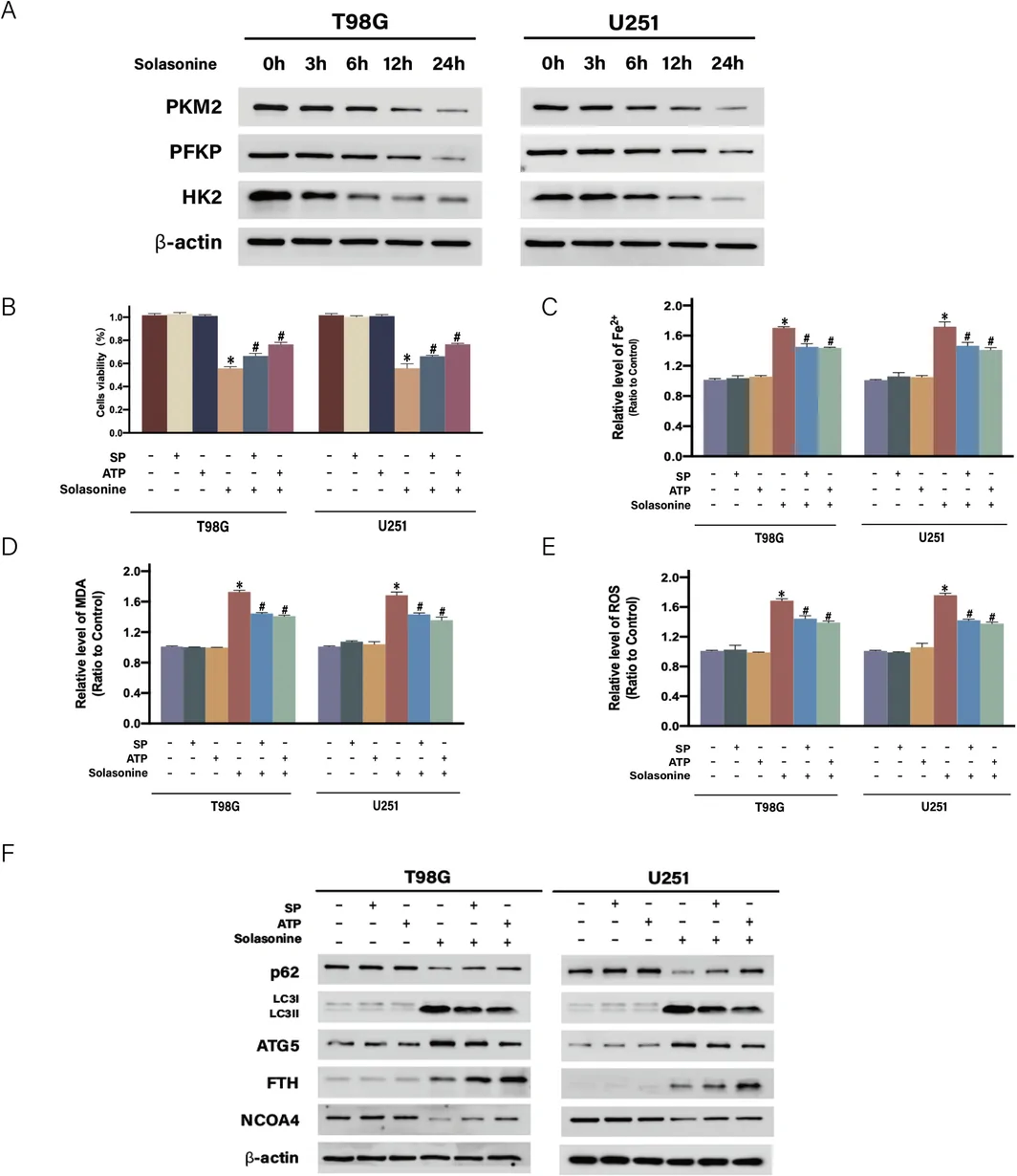
(A) Western blots of glycolysis‐related protein expression after SS treatment. (B–E) ROS, MDA, and ferrous ion levels in SS‐treated cells pretreated with SP and ATP. (F) Western blots of ferritinophagy‐related protein expression in SS‐treated cells pretreated with SP and ATP. *p < 0.01 versus control, #*p* < 0.01 versus treatment group.

The GPX4, xCT, and catalase levels decreased, reflecting an increased hydrogen peroxide level, with prolonged SS treatment (Figure 4A). Direct measurement confirmed that the hydrogen peroxide level increased after SS treatment and decreased with SP and ATP pretreatment (Figure 4B), and thus that the increase was related to the inhibition of glycolysis. The SP and ATP pretreatments also inhibited the SS‐induced reduction of the GPX4, xCT, and catalase levels, indicating that glycolysis maintains the redox balance by reducing the intracellular hydrogen peroxide level (Figure 4C). Treatment with 250 and 500 μM hydrogen peroxide increased the intracellular ferrous ion level (Figure 4D). Treatment with 500 μM hydrogen peroxide also increased the FTH, LC3‐II, and ATG5 levels and decreased the p62 and NCOA4 levels, reflecting the promotion of ferritinophagy (Figure 4E). NAC inhibited SS‐promoted ferritinophagy (Figure 4F). These results indicate that the inhibition of glycolysis in glioma cells by SS promotes ferritinophagy by promoting the increase of the hydrogen peroxide level.

**FIGURE 4:**
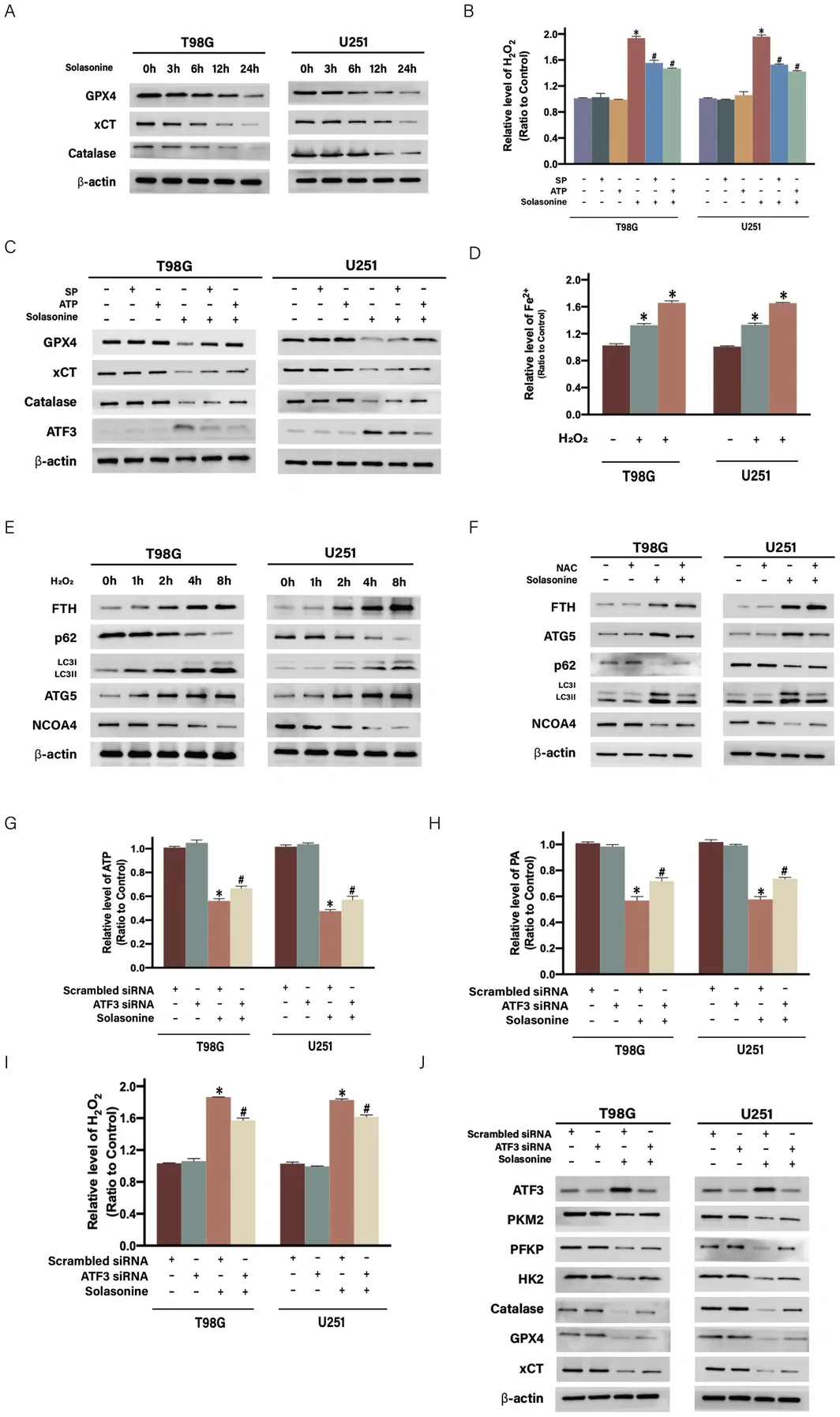
(A) Western blots of antioxidant‐related protein expression after SS treatment. (B, C) The intracellular hydrogen peroxide level and antioxidant‐related protein expression after pretreatment with SP and ATP, respectively, followed by SS treatment. (D) Ferrous ion levels after hydrogen peroxide treatment. (E, F) Western blots of ferritinophagy‐related protein expression in hydrogen peroxide– and NAC‐treated cells. (G–I) ATP, PA, and hydrogen peroxide levels in glioma cells treated with siATF3 and SS. (J) Western blots of glycolysis‐related protein expression in glioma cells treated with siATF3 and SS. *p < 0.01 versus control, #*p* < 0.01 versus treatment group.

ATF3 knockdown increased the intracellular ATP, PA, and hydrogen peroxide levels, suggesting that ATF3 influences the hydrogen peroxide level by regulating glycolysis (Figure 4G–I). It also led to increases in the PKM2, PFKP, and HK2 levels in SS‐treated cells (Figure 4J). In addition, siATF3 treatment reduced the inhibitory effect of SS on GPX4, xCT, and catalase. These results indicate that ATF3 promotes ferritinophagy by inhibiting glycolysis‐related protein expression and increasing the hydrogen peroxide level.

### 3.5 SS Promotes ER Stress Through the Positive Feedback Regulation of ATF3 Expression by Inhibiting Glycolytic Hydrogen Peroxide

The expression of GRP78, PERK, and ATF4 increased with the SS and hydrogen peroxide treatment times (Figure 5A,C), reflecting the promotion of ER stress activation. The SP and ATP pretreatments inhibited the SS‐induced ER stress response (Figure 5B). These results show that the inhibition of glycolysis in glioma cells treated with SS increases the intracellular hydrogen peroxide level, thereby activating ER stress. 4‐PBA inhibited the SS‐induced elevation of the ferrous ion, MDA, ROS, and ferroptosis‐related protein (transferrin, TfR, and ferritin) levels and the reduction of the GPX4 and xCT levels (Figure 5D–G), indicating that it is involved in SS‐induced glioma cell ferroptosis. In addition, 4‐PBA inhibited the SS‐induced elevation of GRP78, PERK, ATF4, and ATF3 expression (Figure 5H), indicating that SS promotes ER stress through the positive feedback regulation of ATF3 expression by inhibiting glycolytic hydrogen peroxide.

**FIGURE 5:**
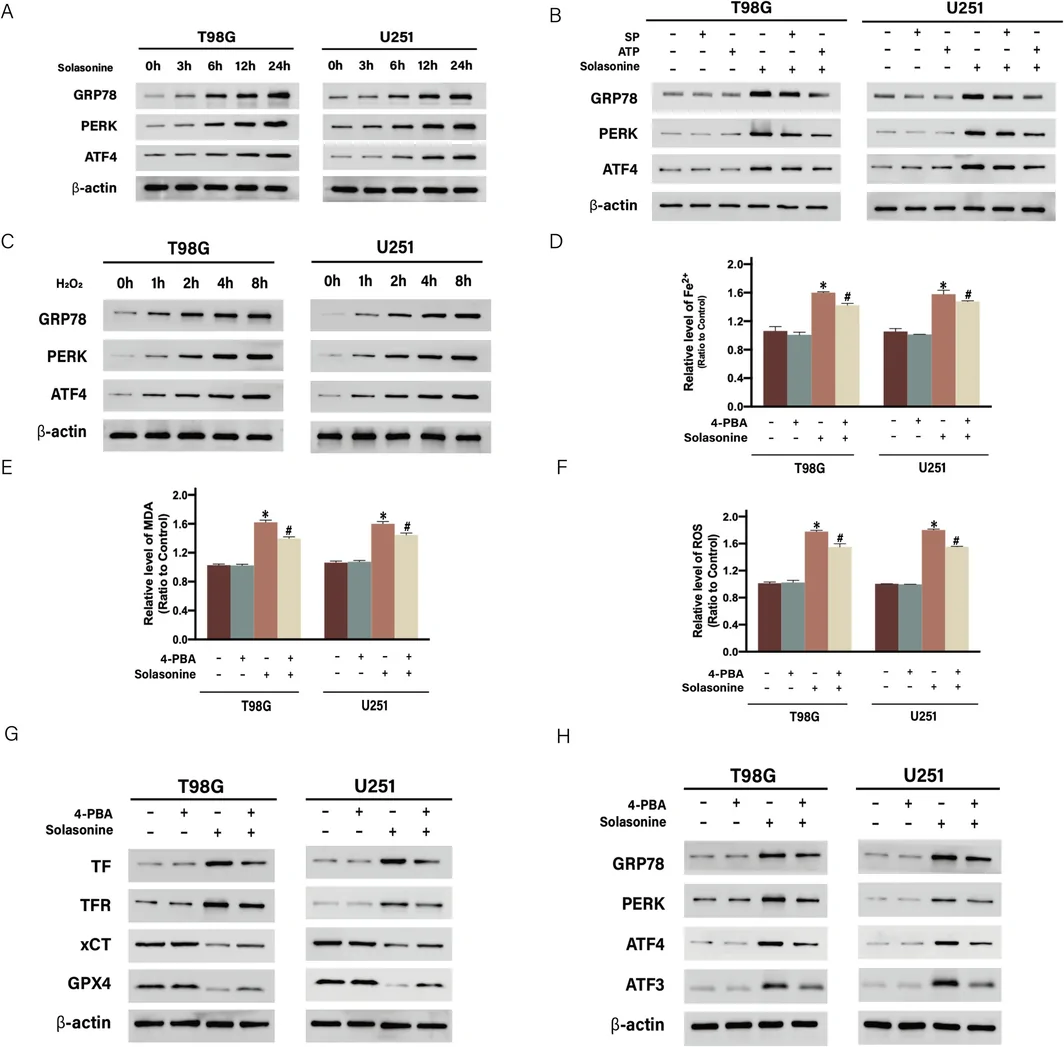
(A–C) Western blots of ER stress–related protein expression after SS treatment with and without SP and ATP pretreatment, and after hydrogen peroxide treatment. (D–F) ROS, MDA, and ferrous ion levels in cells treated with SS after pretreatment with 4‐PBA. (G, H) Western blots of ferroptosis‐ and ER stress–related protein expression in cells treated with 4‐PBA and SS. *p < 0.01 versus control, #*p* < 0.01 versus treatment group.

### 3.6 In Vivo Results

In the murine glioma model, tumor growth was significantly inhibited in the SS‐treated groups compared with that in the control group (Figure 6A,B). The MDA, ferrous ion, and hydrogen peroxide levels were higher and the GSH/GSSG level was lower with the high dose than with the low dose of SS (Figure 6C–F), indicating that SS promotes the ferroptosis of glioma cells in vivo. The expression of transferrin, TfR, ferritin, ATF3, LC3‐II, GRP78, PERK, and ATF4 increased and that of GPX4, xCT, p62, NCOA4, PKM2, HK2, and PFKP decreased with the high‐dose treatment (Figure 6G), indicating that SS inhibits glycolysis through ATF3, increases the hydrogen peroxide level, promotes iron autophagy, and activates ER stress in vivo. H&E staining results demonstrated that, in the mouse intracranial tumor model, tumor growth in the ss‐treated group was significantly inhibited compared to the control group (Figure 6H). Immunohistochemical results revealed that, in the SS‐treated group, the expression of NCOA4, GPX4, and PKM2 was significantly decreased; these findings indicate that this mechanism is also operative within the orthotopic intracranial tumor model (Figure 6I).

**FIGURE 6:**
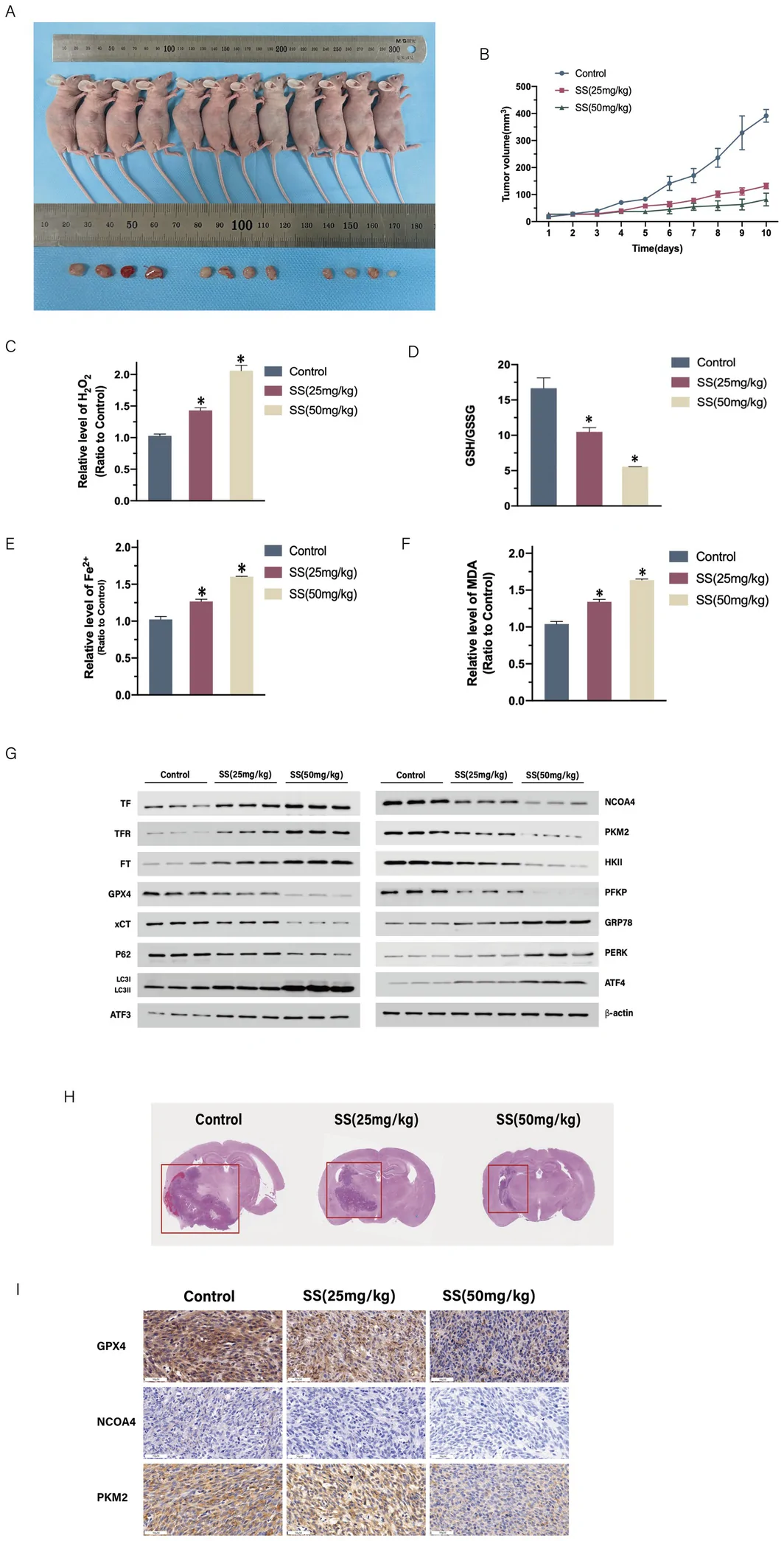
(A) Photograph of nude mouse tumors. (B) Tumor volume over time. (C–F) Hydrogen peroxide, MDA, ferrous ion, and GSH/GSSG levels in tumor tissues from nude mice treated with high and low doses of SS. (G) Western blots of glycolysis‐, ferritinophagy‐, and ferroptosis‐related protein expression in tumor tissues. (H) H&E staining of intracranial orthotopic tumor following SS treatment. (I) Expression of intratumoral NCOA4, PKM2, and GPX4 in each treatment group (bar = 50 μm). *p < 0.01 versus control.

## 4 Discussion

This study showed that SS inhibits glioma proliferation in a dose‐dependent manner, laying the foundation for further research on the mechanism underlying its induction of glioma cell death. In brief, SS promotes lipid peroxidation and causes ferroptosis by increasing iron ion levels and inhibiting antioxidant protein expression to increase the ROS level. It significantly upregulates the expression of ATF3, a key protein in ferroptosis, in glioma cells. ATF3 knockdown ameliorates SS‐induced changes in ferroptosis marker expression and reduces the intracellular iron ion level and accumulation of lipid peroxidation products, indicating that ATF3 plays a key role in the regulation of SS‐induced ferroptosis in glioma cells.

ATF3 was initially studied as a stress response gene [32], but it was discovered to play important roles in various cellular metabolic activities, such as glucose, lipid, and amino acid metabolism [33, 34, 35]. This study showed that ATF3 inhibits glycolysis in glioma cells by reducing the expression of PKM2, PFKP, and HK2. During ferroptosis, cells rely more heavily on glycolysis for energy. This metabolic reprogramming leads to the increased production of pyruvate, ATP, and lactate, which in turn affects the intracellular redox state [36]. Our results suggest that the upregulation of ATF3 inhibits glycolysis, leading to the sustained accumulation of hydrogen peroxide, which impacts oxidative stress and iron metabolism. Furthermore, they suggest that SS interferes with cellular iron uptake, storage, and utilization, increasing intracellular free iron levels and, in turn, promoting lipid peroxidation and triggering ferroptosis. Ferrous ion levels are increased not only by ATF3's regulation of the transferrin and TfR levels, but also via ferritinophagy [37, 38, 39]. A complex interplay exists between autophagy and ferroptosis, depending on the cellular physiological state and stress conditions. On one hand, autophagy can promote ferroptosis by releasing free iron ions and increasing intracellular iron levels through ferritinophagy. On the other hand, autophagy can inhibit ferroptosis by reducing ROS production through the clearance of damaged mitochondria (mitophagy). Furthermore, autophagy can indirectly promote ferroptosis by degrading antioxidant enzymes such as GPX4 [40, 41, 42].

Our results show that ATF3 activates ferritinophagy through the ROS hydrogen peroxide. ATG5 is a key protein in the lipidation of LC3, promoting the formation of LC3‐II. NCOA4 binds to LC3‐II and delivers ferritin to the newly formed autophagosome membrane. p62 can bind to NCOA4, stabilize the NCOA4–ferritin complex, and promote the autophagic degradation of ferritin. ATF3 regulates ferritinophagy through ATG5 to increase the intracellular ferrous ion level, thereby promoting glioma cell ferroptosis. ER stress indirectly promotes ferroptosis by increasing ROS levels. Elevated intracellular concentrations of hydrogen peroxide directly damage the ER membrane structure and function, preventing normal protein folding and thereby triggering ER stress. Hydrogen peroxide can activate multiple ER stress–inducing pathways, such as the inositol‐requiring enzyme 1α–X‐box binding protein 1, PERK‐ATF4, and ATF6 pathways. ATF3 is a target gene of the PERK/ATF4 pathway [43, 44, 45, 46, 47]. Our results indicate that hydrogen peroxide activates the GRP78, PERK, and ATF4 pathways through ER stress, increasing the ATF3 level. The entire process forms a positive feedback loop leading to the ferroptosis of glioma cells.

As a naturally occurring steroidal glycoalkaloid, SS possesses the multifaceted advantages characteristic of natural products, including widespread availability, controllable production costs, multi‐target activity that mitigates the development of drug resistance, and a chemical structure amenable to modification and optimization. Moving forward, its druggability can be further enhanced through strategies such as the utilization of nanodelivery systems and sustained‐release formulations. In the context of glioma treatment, SS serves as a novel inducer of ferroptosis; specifically targeting patients with recurrent or refractory gliomas, it overcomes temozolomide resistance by bypassing conventional mechanisms of apoptosis resistance. Furthermore, it can be administered in combination with radiotherapy to enhance therapeutic sensitivity, while its broad‐spectrum antitumor potential also warrants significant attention.

## 5 Conclusion

This study elucidated the mechanism by which SS induces ferroptosis in glioma cells through ATF3, providing new targets and strategies for glioma treatment. Additional research is needed to validate the findings of this study and further explore their implications. Drug development efforts are needed to improve the bioavailability and therapeutic efficacy of SS through structural modification and drug delivery system design. Furthermore, given the complexity and heterogeneity of gliomas, SS may need to be combined with other treatments, such as surgery, radiotherapy, and chemotherapy, to achieve optimal therapeutic effects. Such combination approaches need to be explored to provide more effective, comprehensive treatment options for patients with glioma.

## Author Contributions

**Mingbo Jia:** data acquisition, statistical analysis, and manuscript preparation. **Hexin Xiao:** data analysis and manuscript preparation. **Minghao Shi:** literature search. **Yang Yang:** manuscript editing. **Jianing Zhao:** manuscript editing. **Zhuoyue Shan:** manuscript editing. **Pengfei Ge:** study design and manuscript editing. **Liyan Zhao:** project administration and manuscript review. All authors have read and approved the final submission.

## Funding

This research was supported by Jilin Provincial Science and Technology Department (20240601004RC).

## Ethics Statement

This animal study was approved by the Animal Care and Use Committee of the First Hospital of Jilin University (Changchun, China) with procedure number 20241230‐01and 20260420‐08.

## Conflicts of Interest

The authors declare no conflicts of interest.

## Data Availability

The date used to support the findings of this study are available from the corresponding author upon request.
